# FBPA PET in boron neutron capture therapy for cancer: prediction of ^10^B concentration in the tumor and normal tissue in a rat xenograft model

**DOI:** 10.1186/s13550-014-0070-2

**Published:** 2014-12-20

**Authors:** Kohei Hanaoka, Tadashi Watabe, Sadahiro Naka, Yasukazu Kanai, Hayato Ikeda, Genki Horitsugi, Hiroki Kato, Kayako Isohashi, Eku Shimosegawa, Jun Hatazawa

**Affiliations:** Department of Nuclear Medicine and Tracer Kinetics, Osaka University Graduate School of Medicine, Suita, Japan; Department of Molecular Imaging in Medicine, Osaka University Graduate School of Medicine, Suita, Japan; PET Molecular Imaging Center, Osaka University Graduate School of Medicine, Suita, Japan; Osaka University Hospital, Suita, Japan; Immunology Frontier Research Center, Osaka University, Suita, Japan

**Keywords:** Boron nuclear capture therapy, Boron concentration, FBPA, BPA-fr

## Abstract

**Background:**

Boron neutron capture therapy (BNCT) is a molecular radiation treatment based on the ^10^B (n, α) ^7^Li nuclear reaction in cancer cells, in which delivery of ^10^B by 4-borono-phenylalanine conjugated with fructose (BPA-fr) to the cancer cells is of critical importance. The PET tracer 4-borono-2-^18^ F-fluoro-phenylalanine (FBPA) has been used to predict the accumulation of BPA-fr before BNCT. However, because of the difference in chemical structure between BPA-fr and FBPA and the difference in the dose administered between BPA-fr (therapeutic dose) and FBPA (tracer dose), the predictive value of FBPA PET for BPA-fr accumulation in the tumor and normal tissues is not yet clearly proven. We conducted this study to validate FBPA PET as a useful test to predict the accumulation of BPA-fr in the tumor and normal tissues before BNCT.

**Methods:**

RGC-6 rat glioma cells (1.9 × 10^7^) were implanted subcutaneously in seven male F344 rats. On day 20 after the tumor implantation, dynamic PET scan was performed on four rats after injection of FBPA for 1 h. Whole-body PET/CT was performed 1 h after intravenous injection of the FBPA solution (30.5 ± 0.7 MBq, 1.69 ± 1.21 mg/kg). PET accumulation of FBPA in the tumor tissue and various normal tissues was estimated as a percentage of the injected dose per gram (%ID/g). One hour after the PET/CT scan, BPA-fructose (167.32 ± 18.65 mg/kg) was injected intravenously, and the rats were dissected 1 h after the BPA-fr injection. The absolute concentration of ^10^B in the autopsied tissues and blood was measured by inductively coupled plasma optical emission spectrometry (ICP-OES).

**Results:**

The highest absolute concentration of ^10^B determined by ICP-OES was found in the kidney (4.34 ± 0.84 %ID/g), followed by the pancreas (2.73 ± 0.63 %ID/g), and the tumor (1.44 ± 0.44 %ID/g). A significant positive correlation was found between the accumulation levels of BPA-fr and FBPA (*r* = 0.91, *p* < 0.05).

**Conclusions:**

FBPA PET can reliably predict accumulation of BPA-fr in the tumor as well as normal tissues.

**Electronic supplementary material:**

The online version of this article (doi:10.1186/s13550-014-0070-2) contains supplementary material, which is available to authorized users.

## Background

Boron neutron capture therapy (BNCT) is based on the nuclear capture reaction of ^10^B (n, α) ^7^Li by low-energy neutrons produced by a nuclear reactor or more recently accelerator. High-energy α particles and lithium ions have been shown to exert a cell-killing effect. In the presence of ^10^B specifically in cancer cells, these particles have been demonstrated to exert a cancer-cell-specific killing effect, because of the short track ranges of these particles (9 to 10 μm for α particles and 4 to 5 μm for ^7^Li nuclei) [1]-[3]. In BNCT, the requisite concentration of ^10^B in the tumor has been estimated to be 15 ppm or more [2]. It is also important to estimate the ^10^B concentration in normal tissues/organs to avoid radiation injury to normal tissues [4]-[6].

In the present BNCT practice, l-paraboronophenylalanine labeled with ^10^B and conjugated with fructose (BPA-fr) is mainly used as the carrier of ^10^B into the tumor cells. In order to evaluate BPA-fr accumulation in the tumors, 4-borono-2-^18^ F-fluoro-phenylalanine (FBPA) PET has been employed [4]. Imahori et al. demonstrated that both BPA-fr and FBPA accumulated in high concentration in high-grade gliomas [5]. In clinical practice, the measurement of FBPA accumulation was made about 1 h after FBPA administration [7]-[10]. However, there are several limitations of FBPA PET in predicting BPA-fr accumulation in the tumors and normal tissues. Firstly, the chemical structure of FBPA differs from that of BPA-fr. Secondly, FBPA PET provides tracer-dose pharmacokinetics of FBPA, while therapeutic doses of ^10^BPA are administered (approximately 500 mg/kg) for BNCT. Thirdly, BPA-fr is administered by slow bolus intravenous injection followed by drip infusion during neutron irradiation, while FBPA is administered by a single bolus injection. Because of these differences, the predictive value of FBPA PET for BPA-fr accumulation in the tumor and normal tissues remains unclear.

In the preset experiment, we first measured the radioactivity accumulation in transplanted tumors and normal organs in rats by means of PET/CT carried out after administration of a tracer dose of FBPA. We then administered a therapeutic dose of BPA-fr and quantified the absolute concentration of ^10^B in autopsy specimens by means of inductively coupled plasma optical emission spectrometry (ICP-OES). The correlations between the ^10^B concentrations after BPA-fr injection and the uptake values of FBPA in FBPA PET were examined in the tumors and normal organs.

## Methods

### Synthesis of l-[^18^F] FBPA

FBPA was prepared as described previously [4], although with several modifications, using an F-1 synthesizer (Sumitomo Heavy Industries, Tokyo, Japan). In brief, ^18^ F-acetylhypofluorite was bubbled at a flow rate of 600 mL/min at room temperature into 5 mL of trifluoroacetic acid containing 30 mg of 4-borono-l-phenylalanine. Next, trifluoroacetic acid was removed by passing N_2_ under reduced pressure at a flow rate of 200 mL/min. The residue was dissolved in 3 mL of 0.1% acetic acid, and the solution was applied to YMC-Pack ODS-A, a high-performance liquid chromatography column (20 mm in inner diameter × 150 mm in length; YMC, Kyoto, Japan), under the following conditions: mobile phase, 0.1% acetic acid; flow rate, 10 mL/min; ultraviolet detector at 280 nm; and radioactivity detector. The FBPA fraction (retention time = 19 to 21 min) was collected. After drying of the FBPA fraction, the residue was dissolved in saline. The radiochemical purity of FBPA was >98%, and the specific activity at the end of the synthesis was 49.7 ± 17.3 GBq/mmol as determined by HPLC.

### Preparation of the BPA-fructose complex

BPA was solubilized at neutral pH for intravenous infusion by allowing it to form a complex with fructose. Injection solutions of the BPA-fructose complex (BPA-fr) were prepared at a concentration of 10 mg BPA/0.43 mL using a previously published procedure with modifications [6].

### Glioma tumor model preparation

Seven male F344/NJcl-rnu/rnu rats (11 to 13 weeks; 241.7 ± 28.0 g) obtained from CLEA Japan (Tokyo, Japan) were used for this study. The RGC-6 rat glioma cell was obtained from RIKEN BRC (Tsukuba, Japan) through the National Bio-Resource Project of MEXT, Japan. RGC-6 cells (1.9 × 10^7^) were implanted subcutaneously as a cell/Matrigel mixture into the backs of F344 rats. On day 20 after the tumor implantation, the rats were starved for 8 h. The animal studies were conducted with the approval of the Animal Care and Use Committee of Osaka University.

### FBPA PET/CT procedure

Rats anesthetized by inhaled 2% isoflurane plus 100% oxygen at 2 L/min were intravenously injected with FBPA at a dose of 30.5 ± 0.7 MBq (1.69 ± 1.21 mg/kg body weight) [11]. To minimize the influence of BPA-fr accumulation from FBPA, the dose of FBPA was set as tracer dose. Then, the rats were imaged with micro PET/CT (Inveon; Siemens Medical Solutions, Knoxville, TN, USA) in a prone position. In four of seven rats, dynamic images were obtained during 1 h (30 frames of 2 min) after the injection. To measure FBPA in comparison with BPA-fr quantification, static images were obtained in seven rats 1 h after the injection for 10 min (5 min/per bed position, two bed positions). All PET images were reconstructed by 2D ordered-subset expectation maximization (16 subsets, 4 iterations) with a 128 × 128 pixel image matrix. The spatial resolution at the center of the field of view (FOV) was 1.62 mm [12]. The CT images were acquired at a tube voltage of 80 kVp and tube current of 140 μA for scatter and attenuation correction. The injected radioactivity of FBPA was measured by a well-type scintillation counter (BeWell; Molecular Imaging Labo, Osaka, Japan). Regions of interest (ROIs) were placed over the tumor, brain, lung, liver, spleen, pancreas, small intestine, large intestine, kidney, and blood pool in the left ventricle on the decay-corrected PET images with reference to CT images. The maximum and average counts in the voxel were automatically converted to radioactivity per milliliter (Bq/mL) by the cross-calibration factor in the PET reconstruction process. Time-activity curves were plotted for both the tumor and the normal tissues, excluding the spleen and intestine. Based on the assumption that tissue density is 1 g/mL, values were converted to radioactivity per gram of tissue (Bq/g). The percentage of the injected dose per gram of tissue (%ID/g) was determined by dividing the radioactivity per gram (Bq/g) by the injected amount of radioactivity (Bq).

### ^10^B assay in RGC-6 glioma-bearing F344 rats after BPA-fr injection

One hour after the PET/CT scan, 40 mg BPA-fr (167.32 ± 18.65 BPA mg/kg body weight) was injected bolus through the tail vein of each rat. The rats were sacrificed 1 h after the BPA-fr injection, and the following tissue samples were collected: tumor, brain, lung, liver, spleen, pancreas, small intestine, large intestine, kidney, and blood. The absolute ^10^B concentrations in the tissue samples were measured by ICP-OES (Vista-MPX ICP-OES spectrometer, Seiko Instruments, Chiba, Japan) [13],[14]. The concentrations of ^10^B from BPA-fr in the normal tissues and tumor tissues were normalized to %ID/g. A part of the tumor tissue was stained with hematoxylin and eosin for light microscopy examination.

### Data analysis

The concentrations of FBPA and BPA-fr in the tissues, which were measured by PET/CT and ICP-OES, respectively, were shown as mean ± SD. The Pearson product-moment correction coefficients were calculated to evaluate the correlations between the FBPA accumulations measured by PET/CT and BPA-fr accumulations measured by ICP-OES. Wilcoxon's signed-rank test was performed to compare the accumulation level of FBPA estimated by PET/CT and accumulation level of BPA-fr measured by ICP-OES in each of the tissues. All the statistical analyses were performed with the SPSS software (Version 17, SPSS Inc., Chicago, IL, USA), and a *p* value of less than 0.05 indicated a significant difference.

## Results

Table 1 shows absolute values of boron concentrations measured by ICP-OES in the tissues. The highest concentration was found in the kidney, followed by that in the pancreas and the glioma tumor tissue.

**Table 1:** **Absolute of boron concentration after injection of 40 mg (167.32 ± 18.65 mg/kg body weight) BPA by ICP-OES (ppm)**

	Absolute of boron concentration (ppm)	
	Median	Mean ± SD
Tumor	24.31	27.56 ± 8.42
Brain	7.73	7.66 ± 1.15
Lung	17.76	15.12 ± 3.83
Liver	13.80	14.93 ± 2.49
Spleen	19.04	20.48 ± 4.59
Pancreas	50.66	52.25 ± 12.06
Small intestine	16.45	16.46 ± 2.49
Large intestine	12.97	13.21 ± 1.53
Kidney	77.64	83.06 ± 16.08
Blood	12.44	12.25 ± 0.96

**Figure 1:**
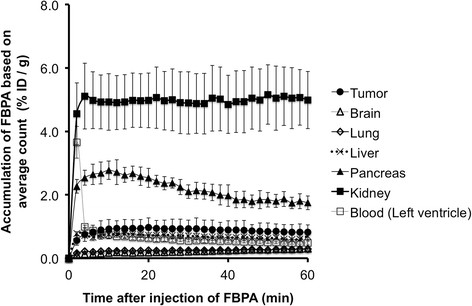
**Time-activity curve.** Based on average count of tumor and normal organs in F344 rats after administration of FBPA.

Figure 1 shows the time-activity curves based on average count of the tumor and several organs in F344 rats after administration of FBPA. Time-activity curves of the tumor and normal tissues showed various patterns of rapidly increasing FBPA uptake up to 20 min, which stabilized or decreased thereafter gradually. Dynamic PET data demonstrated the ratio of tumor to blood (1.24, 1.67, and 1.71) at 10, 30, and 60 min after administration of FBPA, respectively.

**Figure 2:**
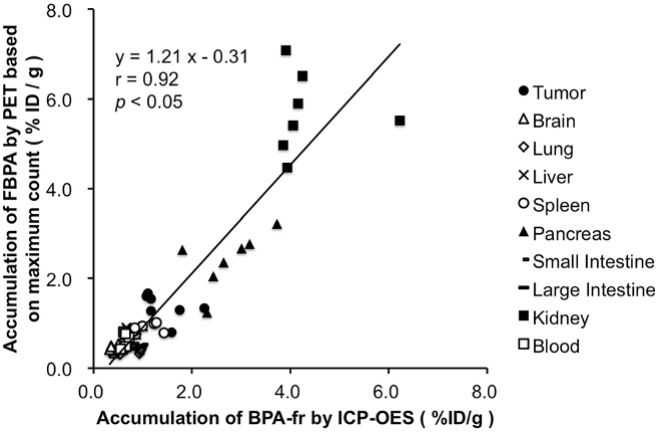
**Correlation between accumulation levels based on maximum count.** Correlation between the accumulation level of BPA-fr measured by ICP-OES and that of FBPA estimated by FBPA PET/CT based on the maximum count in a rat model.

**Figure 3:**
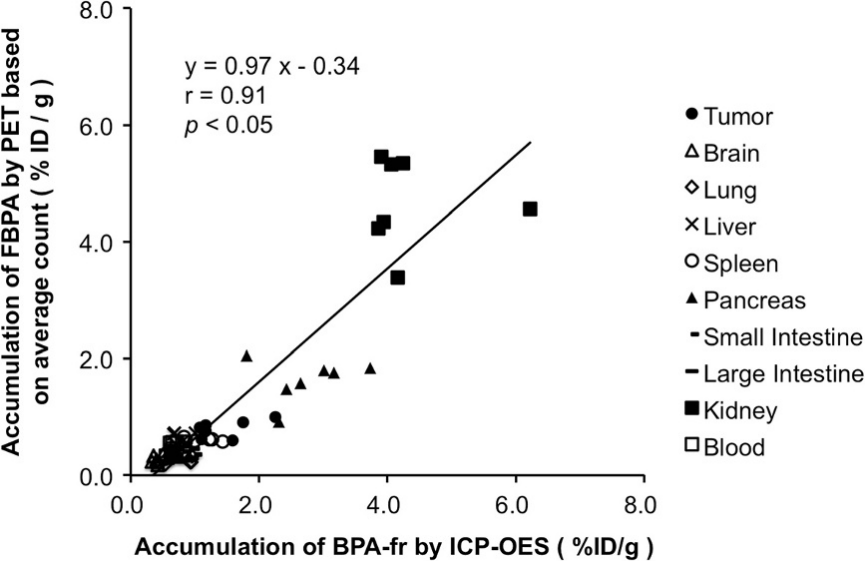
**Correlation between accumulation levels based on average count.** Correlation between the accumulation level of BPA-fr measured by ICP-OES and that of FBPA estimated by FBPA PET/CT based on the average count in a rat model.

The accumulation levels of FBPA measured by PET/CT and expressed as %ID/g demonstrated significant positive correlations with the accumulation levels of BPA-fr measured by ICP-OES and expressed as %ID/g; the regression lines were as follows: *y* = 1.21x − 0.31 (*r* = 0.92, *p* < 0.05) for FBPA accumulation based on the maximum count and *y* = 0.97x − 0.34 (*r* = 0.91, *p* < 0.05) for FBPA accumulation based on the average count (Figures 2 and 3).

**Figure 4:**
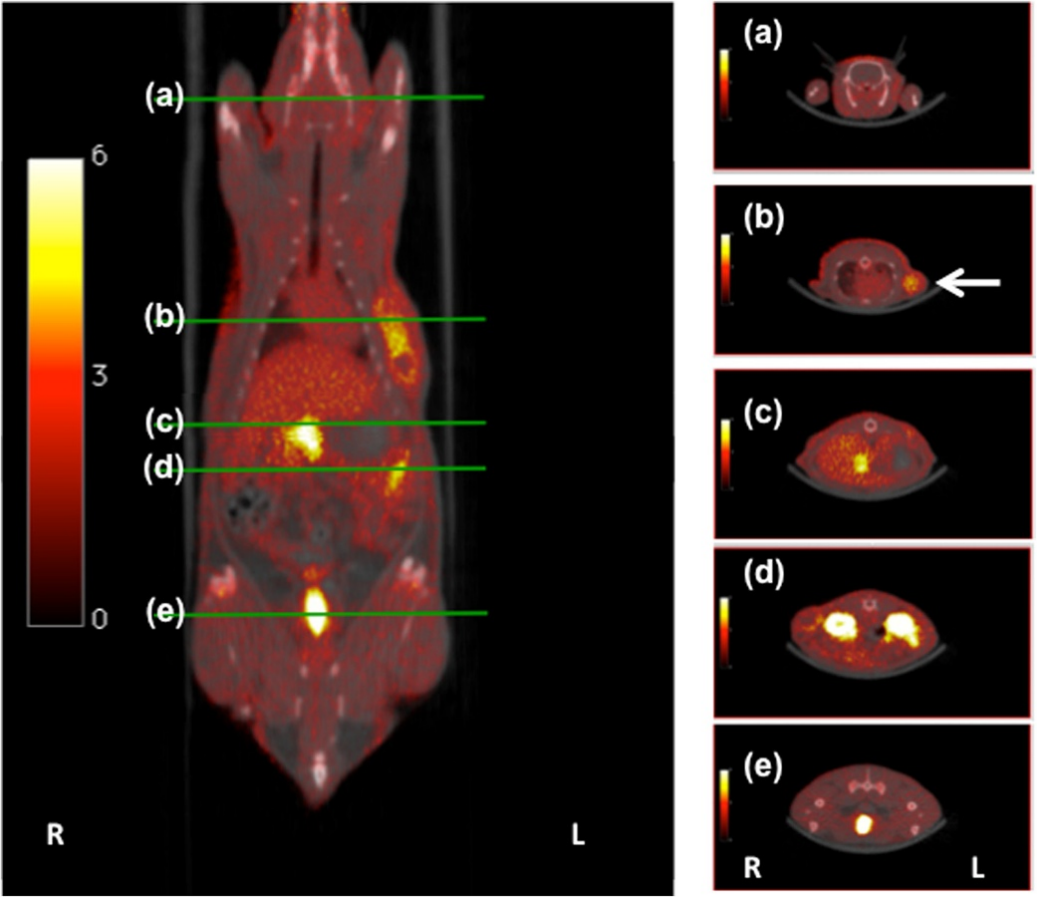
**PET/CT images of a RGC-6 glioma-bearing F344 rat.** PET/CT fused images of F344 male rats with RGC-6 glioma xenografts 60 min after injection of approximately 30 MBq of FBPA. **(a)** At the level of brain, **(b)** at the level of heart and transplanted tumor, **(c)** at the level of pancreas and liver, **(d)** at the level of kidneys, and **(e)** at the level of bladder (all transaxial images). FBPA accumulation in the tumor was found in the plane **(b)** indicated by an arrow. In the planes **(c)**, **(d)**, and **(e)**, high accumulations were found in the pancreas, kidneys, and bladder, respectively.

Figure 4 depicts the FBPA PET/CT images of a RGC-6 glioma-bearing F344 rat. Significant accumulation in the tumor and high radioactivity contrast between the tumor and normal tissues are observed. High uptakes of FBPA in the kidneys and pancreas of the rats are seen.

Table 2 shows the accumulation levels of FBPA measured by PET/CT and accumulation levels of BPA-fr measured by ICP-OES, expressed as %ID/g of tissue. The estimated values by FBPA PET based on the maximum count in the blood, brain, liver, pancreas, and tumor were similar to the values measured by ICP-OES. The differences did not exceed 15% in any of these tissues. In the lung, small intestine, and large intestine, the FBPA accumulation measured by FBPA PET based on the maximum count and average count was significantly underestimated. In the tumor, the value based on the average count was significantly underestimated. In the kidney, the value based on the maximum count was significantly overestimated.

**Table 2:** **Biodistribution of BPA-fr and [**^**18**^**F] FBPA in various organs of RGC6 glioma-bearing Fischer 344 rats (** ***n***  **= 7)**

	Accumulation of BPA-fr by ICP-OES (%ID/g)		Accumulation of FBPA by PET based on maximum count (%ID/g)		Accumulation of FBPA by PET based on average count (%ID/g)	
	Median	Mean ± SD	Median	Mean ± SD	Median	Mean ± SD
Tumor	1.27	1.44 ± 0.44	1.34	1.36 ± 0.31	0.80	0.79 ± 0.18*
Brain	0.40	0.40 ± 0.06	0.41	0.43 ± 0.07	0.26	0.27 ± 0.07*
Lung	0.93	0.79 ± 0.20	0.39	0.38 ± 0.05*	0.23	0.25 ± 0.05*
Liver	0.72	0.78 ± 0.13	0.78	0.78 ± 0.14	0.59	0.61 ± 0.12
Spleen	0.99	1.07 ± 0.24	0.89	0.88 ± 0.10	0.59	0.58 ± 0.08*
Pancreas	2.65	2.73 ± 0.63	2.64	2.42 ± 0.63	1.76	1.63 ± 0.36*
Small intestine	0.86	0.86 ± 0.13	0.48	0.52 ± 0.10*	0.36	0.36 ± 0.08*
Large intestine	0.68	0.69 ± 0.08	0.44	0.46 ± 0.09*	0.30	0.30 ± 0.07
Kidney	4.06	4.34 ± 0.84	5.52	5.70 ± 0.89*	4.56	4.66 ± 0.76
Blood	0.65	0.64 ± 0.05	0.59	0.61 ± 0.14	0.45	0.47 ± 0.10*

*p < 0.05 compared to BPA-fr by ICP-OES.

**Figure 5:**
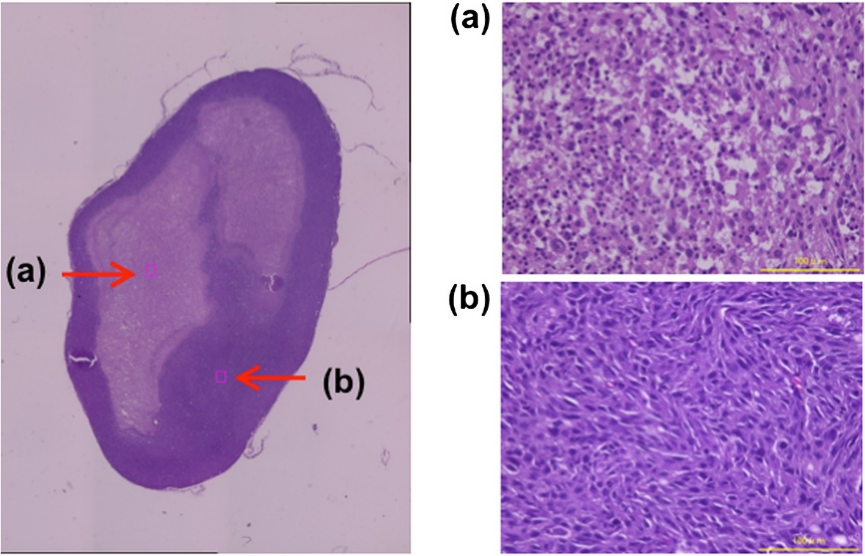
**Representative light microscopy images of the glioma (hematoxylin-eosin staining).** Area **(a)** has a lower cell density than area **(b)**, indicating the heterogeneity of the tumor components.

Figure 5 shows a light microscopy image of a hematoxylin-eosin-stained section (scale bars = 100 μm) of the glioma, showing the heterogeneity of the lesion.

## Discussion

In this study, we demonstrated a significant correlation between the concentrations of ^10^B and accumulation levels of FBPA in tumors and various normal organs, despite the differences in the chemical structure and dose administered between BPA-fr and FBPA.

Kabalka et al. reported the optimal time for neutron exposure after infusion of BPA-fr. They reported that the tumor-to-blood activity ratio of humans appeared to plateau after 60 mm [10]. In the present study, the time point of 1 h after administration for FBPA PET may be considered appropriate because the tumor-to-blood ratio becomes stable. More time points are needed for in-depth dosimetric studies. Nevertheless, one time point of 1 h is a good compromise to obtain a quantitative value that can be correlated with other variables.

Yoshimoto et al. recently reported that FBPA is predominantly transported via the l-type amino acid transporter in human glioblastoma cells [15]. They also demonstrated that uptake of FBPA into human glioblastoma cells was inhibited by high concentrations of BPA in the medium, suggesting that FBPA and BPA share the same transporter system. Imahori et al. also reported that the ^10^B concentrations in surgically removed specimens of glioblastoma after administration of BPA-fr at therapeutic doses were predicted by using the rate constants of FBPA PET [16]. In this study, the injected dose of BPA-fr was about 100 times higher than that of FBPA. Notwithstanding, significant correlations were observed between the amounts of BPA-fr and FBPA accumulated in the tumors as well as normal organs. Our results suggest that FBPA PET can be used to estimate the amount of BPA accumulation in the normal surrounding tissues during BNCT. The accumulation levels of both BPA and FBPA in the pancreas were remarkably high (2.73 and 2.42 %ID/g, respectively). This finding suggests that radiation injury of the pancreas should be anticipated when BNCT is employed for abdominal cancers.

There are several reports suggesting similar pharmacokinetics between BPA-fr and FBPA in normal tissues/organs. Wang et al. [17] measured the radioactivity in glioma and normal tissues after injection of FBPA-fr by a γ-scintillation counter and compared the ^10^B concentrations by ICP-MS after injection of BPA-fr as the tumor-to-normal tissue ratio. The tumor-to-normal tissue uptake ratio of FBPA-fr was parallel to that of BPA-fr. Yang et al. [18],[19] reported the pharmacokinetic analysis of FBPA and BPA-fr after ultrasound-induced blood-brain barrier disruption. Ishiwata et al. [20] reported that the ratios of the concentrations of FBPA to those of BPA in the tumor, blood, and muscle measured by a NaI(Tl) gamma counter and ICP-AES, respectively, ranged from 0.70 to 1.00. These reports lend support to our view that FBPA PET can be used for the assessment of BPA accumulation in normal tissues/organs during BNCT in an attempt to avoid radiation injury.

In BNCT, the subcellular location of ^10^B is of critical importance for the cell-killing effect, because the trace ranges of α particles and ^7^Li are very short. Chandra et al. [21] reported that there was no significant difference in the intracellular distribution between FBPA and BPA as assessed by ion microscopy. Therefore, it is expected that FBPA PET would reflect the intracellular distribution of BPA-fr and predict the therapeutic effect of α particles and ^7^Li which have short track ranges.

In our study, underestimations by FBPA PET were observed in the lung and intestines. One of the reasons for this may be the partial volume effect, because of the limit of resolution of PET. The partial volume effect, which occurs at sizes less than or equal to three times the full width at half maximum, was not corrected for this study [22],[23]. The full width at half maximum of the PET system used in this study in axial resolutions at the center of the FOV was 1.62 mm; therefore, differences in the sizes of the tissues may influence the %ID/g. Especially in the lung and intestines, the influence of the partial volume effect is serious, because the regions of interest on the PET images contain air [24].

On the other hand, overestimation was noted in the kidney. FBPA is metabolically stable. During the initial 1 h, about half of the injected amount of tracer was passed into the urine [25]. In the kidney, contamination with the count from the urine contained in the ureter or renal pelvis should be considered on FBPA PET images.

In the tumor, there was a 40% difference between the maximum count and average count on FBPA PET images. The slight overestimation of the accumulation level in the tumor in this study is mainly because gliomas consist of heterogeneous tissue components, including viable portions, central necrosis areas, and peritumoral infiltration areas [26] (Figure 5).

Conventionally, BNCT has been used for the medical treatment of malignant melanoma [27], malignant brain tumors [5],[16],[28]-[30], and cancer of the neck [31]. In recent years, adoption of BNCT has expanded to liver cancer [32], breast cancer, and lung cancer [33]. Therefore, prediction of the absolute concentration of BPA in the normal tissues in individual patients is very important to minimize the radiotoxicity to normal healthy tissues during BNCT.

Our study had some limitations. First, the method of administration of BPA-fr for BNCT is different from the infusion method adopted for administration of FBPA for PET/CT, especially because PET examination requires only a low dose of FBPA. BPA is administered via intravenous infusion. Therefore, determination of the offset according to the infusion method of BPA-fr is required to ensure the accuracy of the clinical data.

Second, the biodistribution of FBPA in animals differs from that in humans [25]. Especially in the pancreas, the expression levels of LAT1 between rats and humans are different. The accumulation levels of FBPA and BPA-fr determined in this research may therefore not be applicable to clinical cases. Further clinical FBPA PET study is required to determine BPA-fr distribution in the cancer patients.

## Conclusions

In this study, we demonstrated the existence of clear correlations between the accumulation levels of BPA-fr and FBPA in the transplanted glioma cells and normal organs in rat xenograft models. This preclinical study indicates the validity of FBPA PET for predicting BPA-fr accumulation in tumors and normal tissues/organs in BNCT. Further studies are required to estimate the ^10^B concentrations in tissues following BPA-fr administration according to a clinical protocol, such as slow infusion and drip infusion of BPA during neutron irradiation.

## Authors’ original submitted files for images

Below are the links to the authors’ original submitted files for images. Authors’ original file for figure 1 Authors’ original file for figure 2 Authors’ original file for figure 3 Authors’ original file for figure 4 Authors’ original file for figure 5
