# Supplementary figures and images for: FBPA PET in boron neutron capture therapy for cancer: prediction of 10B concentration in the tumor and normal tissue in a rat xenograft model
Source: EJNMMI Res. 2014 Dec 20;4:70. doi: 10.1186/s13550-014-0070-2 (PMC4293470; doi:10.1186/s13550-014-0070-2)

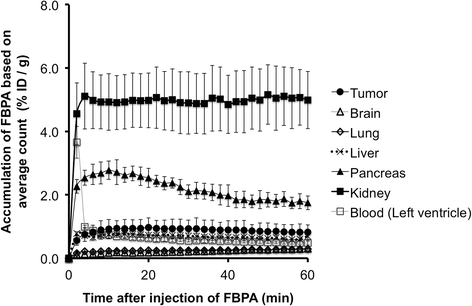

Supplement: Supplementary file 1 — Authors’ original file for figure 1 [file 13550_2014_70_MOESM1_ESM.gif]

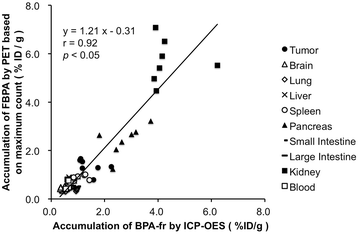

Supplement: Supplementary file 2 — Authors’ original file for figure 2 [file 13550_2014_70_MOESM2_ESM.gif]

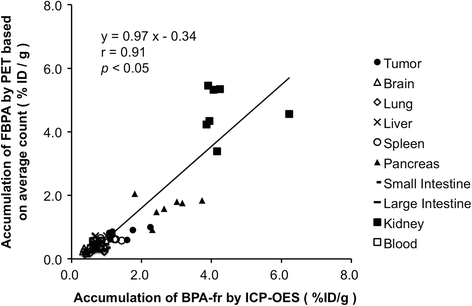

Supplement: Supplementary file 3 — Authors’ original file for figure 3 [file 13550_2014_70_MOESM3_ESM.gif]

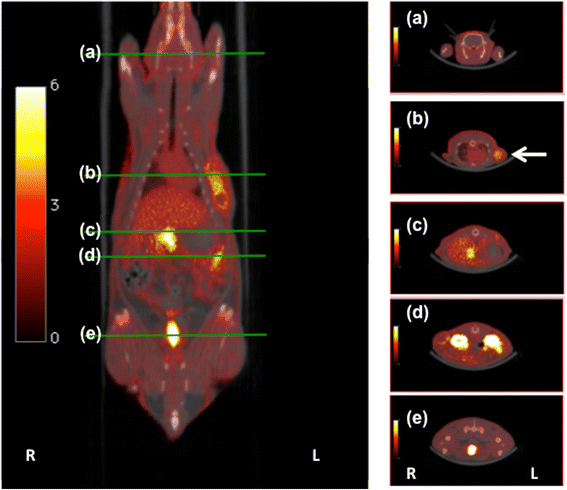

Supplement: Supplementary file 4 — Authors’ original file for figure 4 [file 13550_2014_70_MOESM4_ESM.gif]

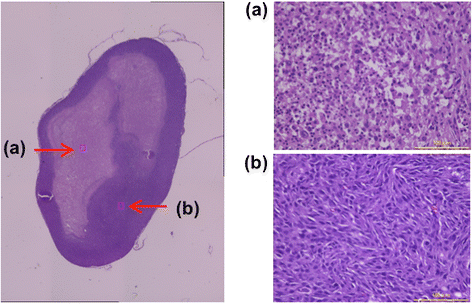

Supplement: Supplementary file 5 — Authors’ original file for figure 5 [file 13550_2014_70_MOESM5_ESM.gif]
